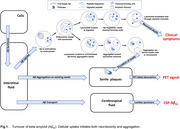# Amyloid Degradation Toxicity Hypothesis: the framework that explains multiple phenomena and paradoxes associated with Alzheimer’s disease

**DOI:** 10.1002/alz70861_108887

**Published:** 2025-12-23

**Authors:** Dmitry V Zaretsky, Maria V. Zaretskaia

**Affiliations:** ^1^ Zarbio Laboratories, Chapel Hill, NC USA; ^2^ Zarbio, Chapel Hill, NC USA

## Abstract

**Background:**

Amyloid Degradation Toxicity Hypothesis proposes that the key etiological molecular event of AD is the membrane channel formation by amyloid fragments produced in lysosomes. The pathogenesis includes lysosomal permeabilization and leakage of lysosomal proteases into the cytoplasm. Within this framework, cellular amyloid uptake initiates both amyloid‐induced cytotoxicity and the appearance of extracellular amyloid aggregates. This explains why AD diagnosis is associated with a high density of non‐toxic amyloid aggregates and lower concentrations of cytotoxic soluble beta‐amyloid. In this presentation, we extend the hypothesis to explain various phenomena and paradoxes associated with AD pathobiology across molecular, cellular, and biomarker levels.

**Method:**

First, we formulated a list of axiomatic statements forming the framework that are considered experimentally proven by the scientific community. Next, we created a list of clinical and experimental phenomena that are associated with Alzheimer’s disease. Finally, we interpreted these phenomena from the perspective of these axiomatic statements. In many instances, the interpretation resulted in novel testable hypotheses, which were formulated as a result.

**Result:**

The hypothesis provided an interpretation for the multiple phenomena including the following:

• the link between AD development and lysosomal failure and mitochondrial dysfunction,

• the extracellular senile plaques co‐labelling with intracellular lysosomal markers,

• why beta‐amyloid is accumulated intracellularly in addition to the extracellular aggregates,

• pathophysiological relevance of increased biomarkers of apoptosis in brains of AD patients,

• temporal dynamics of various biomarkers during AD progression.

The theory provided testable hypotheses on the molecular mechanisms mediating high cytotoxicity of oligomeric forms of beta‐amyloid, as well as the reasons why the recently tested treatments (such as BACE1‐inhibitors and anti‐amyloid antibodies) did not result in prevention of AD progression.

**Conclusion:**

Amyloid Degradation Toxicity Hypothesis is a conceptual framework that is open to developing and changing over time based on new findings relevant to the AD etiology. We expect that the testing of follow‐up hypotheses, which can be formulated based on this framework, will extend our understanding of AD. Notably, the theory suggests new AD‐pathobiology‐relevant biomarkers and novel, previously untested molecular targets for creating pharmacological agents to prevent or slow down the progression of this devastating neurodegenerative disease.